# The BLT Humanized Mouse Model as a Tool for Studying Human Gamma Delta T Cell-HIV Interactions *In Vivo*

**DOI:** 10.3389/fimmu.2022.881607

**Published:** 2022-05-20

**Authors:** Shivkumar Biradar, Yash Agarwal, Michael T. Lotze, Moses T. Bility, Robbie B. Mailliard

**Affiliations:** ^1^Department of Infectious Diseases and Microbiology, Graduate School of Public Health, University of Pittsburgh, Pittsburgh, PA, United States; ^2^Department of Surgery, University of Pittsburgh School of Medicine, Pittsburgh, PA, United States; ^3^Department of Immunology, University of Pittsburgh School of Medicine, Pittsburgh, PA, United States; ^4^Department of Bioengineering, University of Pittsburgh School of Medicine, Pittsburgh, PA, United States

**Keywords:** BLT mice, humanized mice, gamma delta T cells, HIV infection, HIV immunopathogenesis

## Abstract

Gamma-delta (γδ) T cells recognize antigens in a major histocompatibility complex (MHC) independent and have cytotoxic capability. Human immunodeficiency virus (HIV) infection reduces the proportion of the Vδ2 cell subset compared to the Vδ1 cell subset of γδ T cells in the blood in most infected individuals, except for elite controllers. The capacity of Vδ2 T cells to kill HIV-infected targets has been demonstrated *in vitro*, albeit *in vivo* confirmatory studies are lacking. Here, we provide the first characterization of γδ T cell-HIV interactions in bone marrow-liver-thymus (BLT) humanized mice and examined the immunotherapeutic potential of Vδ2 T cells in controlling HIV replication *in vivo*. We demonstrate a reduced proportion of Vδ2 T cells and an increased proportion of Vδ1 T cells in HIV-infected BLT humanized mice, like in HIV-positive individuals. HIV infection in BLT humanized mice also impaired the *ex vivo* expansion of Vδ2 T cells, like in HIV-positive individuals. Adoptive transfer of activated Vδ2 T cells did not control HIV replication during cell-associated HIV transmission in BLT humanized mice but instead exacerbated viremia, suggesting that Vδ2 T cells may serve as early targets for HIV replication. Our findings demonstrate that BLT humanized mice can model γδ T cell-HIV interactions *in vivo*.

## Introduction

Human gamma-delta (γδ) T cells are widely distributed throughout barrier tissues and mediate potent antiviral effects by targeting stressed cells in an MHC-independent manner (1–3). Although human γδ T cells typically makeup <10% of the total T cell population, recognize nonpeptide microbial antigens and play an essential role in controlling various diseases, particularly malaria (4), contributing to both innate and adaptive immune responses (5). While CD4+ T cells are known to be targeted and depleted during the course of HIV infection, there is also a dramatic and immediate impact on γδ T cells, where the normal proportions of the two major subsets of γδ T cells (designated Vδ1 and Vδ2) become inverted due to a selective depletion of Vδ2 T cells expressing the phosphoantigen-responsive Vγ9 chain (Vγ9Vδ2 T cells) (6). Natural history studies of HIV infection demonstrate an inverse correlation between Vδ2 T cell frequency and HIV viral titers (7), and earlier clinical reports indicated that, unlike most HIV-positive individuals, Vδ2 T cells are maintained at a normal frequency in elite controllers (7). The hypothesis for these observations is that γδ T cells may provide protective immunity against HIV infection by secreting chemokines that compete for HIV entry coreceptors or by promoting the effector activity and recruitment of other immune cells to eliminate infected targets. A few *in vitro* studies demonstrated the direct cytotoxic capacity of Vδ2 T cells against HIV-infected targets (8, 9), but the *in vivo* function and therapeutic potential of Vδ2 T cells against HIV has yet to be fully elucidated.

Non-human primate models of the simian immunodeficiency virus (SIV) dominate the current *in vivo* approaches to understanding the relationship between HIV viremia and γδ T cells. However, SIV contains only about 50 percent of the genetic code of HIV, and there are substantial differences in γδ subset composition and phenotype in monkeys and humans (10). The information we can extrapolate from non-human primate models of SIV becomes limited by the unaltered peripheral Vδ1/Vδ2 T cell ratio in SIV-infected macaques (11) and the genetic differences between SIV and HIV (10). Therefore, an alternate approach is needed to understand the *in vivo* dynamics of γδ T cells in HIV infection. Among the widely used *in vivo* platforms for investigating HIV pathogenesis and therapeutics is the mouse model utilizing bone marrow-liver-thymus (BLT) humanized mice (huMice). Generated *via* peripheral injection of CD34+ hematopoietic stem cells (HSCs) and autologous transplantation of fetal liver and thymic explants into immunodeficient mice, BLT huMice provide both the peripheral immune circulation and human lymphoid microenvironment to study HIV in blood and human lymphoid tissues. Previously it has been shown that human CD4^+^/CD8^+^ T cell ratios before and after HIV infection of BLT huMice are comparable to clinical values seen in natural human infection (12). While in humans Vδ2 T cells become depleted during the early stages of natural HIV infection, often before the CD4^+^/CD8^+^ T cell ratio inverts, the impact of HIV infection on γδ T cells has yet to be fully characterized in the BLT huMouse model.

In the present study, we provide the first reported phenotypic and functional characterization of human γδ T cells in BLT huMice and evaluate how they are impacted by HIV infection *in vivo*, and we assess their therapeutic potential following adoptive cell transfer. We demonstrate that the BLT huMouse model recapitulates the clinical changes in Vδ1 and Vδ2 T cell frequencies in the peripheral blood reported during natural HIV infection in humans, providing for the first time an *in vivo* model relevant for studying human γδ T cell biology and γδ T cell-HIV interactions. We used this *in vivo* model to examine the therapeutic impact of adoptively transferred human Vδ2 T cells on cell-associated HIV transmission and replication (13, 14). Surprisingly, the adoptive transfer of allogenic Vδ2 T cells into BLT huMice enhanced, rather than controlled, HIV replication following cell-associated HIV transmission. This escalation in viral production was accompanied by a marked increase in HIV p24-positive Vδ2 T cells in the blood of BLT huMice, suggesting that the Vδ2 T cells may serve as early targets for HIV infection and replication.

## Materials and Methods

### Construction of BLT HuMice

Non-Obese Diabetic. Cg-Prkdcscid Il2rgtm1Wjl/SzJ (NSG) mice were obtained from the Jackson Laboratory and bred in the Division of Laboratory Animal Resources facility at the University of Pittsburgh. The mice were bred and housed under biosafety level 1, pathogen-free conditions according to the guidelines approved by the Institutional Animal Care and Use Committee and were fed irradiated chow (Prolab Isopro RMH 3000 Irradiated, catalog 5P75-RHI-W 22, PMI Nutrition International) and autoclaved water. Human fetal tissues were obtained from the Health Sciences Tissue Bank at the University of Pittsburgh and Advanced Bioscience Resources Inc and processed under biosafety level 2 conditions. Within 12 hours of receiving fetal human liver and thymus, CD34+ hematopoietic stem cells (HSCs) were isolated from the fetal liver as previously described (15) and cryopreserved at -170°C until transplantation. Portions of the fetal liver and thymus tissues were cut into small pieces (<3mm^3) and cryopreserved in Serum-Free Freezing Media (ATCC 30-2600) at -170 C until transplantation. 8 to 10-week-old NSG mice received a radiation dose of 1.50 Gray before transplantation to myoablate the animals and were immediately transferred to biosafety level 2+ animal housing. On the day of operation, the cryopreserved CD34+ HSCs and tissues from two different fetal donors were thawed in a warmed culture medium supplemented with 10% fetal bovine serum. The tissues were minced into ~1-mm^3^ fragments, and the irradiated mice were anesthetized using 1.5-3% isoflurane. Autologous human fetal thymus and liver tissue sections were implanted under the kidney capsule, and 150,000 CD34+ HSCs were engrafted *via* retroorbital injection in a volume of 100 uL. Immediately following the procedure, the mice received 150uL injections of carprofen (1 mg/mL) and ceftiofur (1 mg/mL) as an analgesic and antibiotic, respectively. These injections continued once a day for two days for three sets of injections. Successful engraftment was determined by flow cytometric analysis of human CD45 expression on blood cells of mice, now termed BLT huMice. Mice harboring >30% of human CD45^+^ cells were randomly assigned to groups in further experiments.

### Study Participants

Specimens obtained from participants of the Multicenter AIDS Cohort Study (MACS), now the MACS/WIHS Combined Cohort Study (MWCCS), were used in this study. The contents of this publication are solely the responsibility of the authors and do not represent the official views of the funding sources. The authors express their sincerest gratitude to MWCCS Principal Investigators Dr. Charles R. Rinaldo and Dr. Jeremy Martinson (U01-HL146208), William G. Buchanan, and the participants of the Pittsburgh site of the MWCCS. These participants were HIV-1 infected men who were on ART for a median duration of 12.08 years, who had a median CD4^+^ T cell count of 620 cell/μl and a viral load of <50 copies/ml. Wherever mentioned, blood products from age-matched HIV-negative individuals were used in the study. Whole blood products from HIV-1-seronegative blood donors were purchased from the Central Blood Bank of Pittsburgh. Written informed consent was obtained from participants before inclusion in the study, which was approved by The University of Pittsburgh Institutional Review Board.

### Isolation of Monocytes and Peripheral Blood Lymphocytes

Peripheral blood mononuclear cells (PBMC) were obtained from a buffy coat, or whole blood was isolated by standard density gradient separation using Lymphocyte Separation Medium (Corning). Monocytes were isolated from PBMC by positive magnetic bead selection (Miltenyi Biotec), and CD4+ T cells and γδ T cell subsets (refers to Vδ1 and Vδ2 T cells) were isolated by negative selection (EasySep CD4 T cell, Cat #-17952 and γδ T cell isolation kit, Cat #- 19255) according to the manufacturer’s specifications, and the differentially isolated cells were cultured or cryopreserved until use

### Flow Cytometry

50-100ul of blood was obtained from the submandibular vein of BLT huMice to check for reconstitution and intermediate infection time points. At 4-6 weeks post-HIV/mock infection, BLT huMice were sacrificed, and the entire blood volume was collected *via* orbital bleed. The murine spleen and the transplanted human spleen and thymus were dissected. Excised tissues were homogenized *via* mechanical dissociation, and single-cell suspensions were retrieved after tissue samples were passed through a 100um filter. Red blood cells were lysed and removed from both blood and spleen samples using ACK lysing buffer (Thermo Fisher) as described by the manufacturer before using samples for flow cytometry. Single-cell suspensions prepared from peripheral blood, splenocytes, and thymocytes from each BLT huMouse were stained with a live/dead fixable aqua dead cell stain kit (Thermo Fisher Scientific). For surface staining, cells were preincubated with 1× PBS labeling buffer containing 2% BSA, 0.1% NaN3, and unfractionated murine IgG (1.0 μg/mL; Sigma-Aldrich Cat# 15381-1MG) to block Fc-receptor binding. Then stained the cells with fluorochrome-conjugated antibodies [anti-human CD45, anti-human CD4, anti-human Vδ2, (BioLegend); anti-human CD8, CD3, PD1, HLA-DR, CD25, CD69, CD45RA, and CD27 (Becton Dickenson); and anti-human Vδ1, (Thermo Fisher Scientific)] and intracellular staining with HIV-p24 (KC57, Beckman Coulter). Cells were fixed using 2% paraformaldehyde, and data were acquired using an LSR Fortessa flow cytometer (BD Biosciences) and analyzed using FlowJo software. Gating was done based on Fluorescence minus one (FMO).

### Immunohistochemistry

Paraffin-embedded fixed sections were stained with indicated anti-human antibodies (Anti-human TCR δ monoclonal IgG1 κ antibody, Clone H-41, catalog number sc-100289, Santa Cruz Biotechnology; Ultra-LEAF™ Purified Mouse IgG1, κ Isotype Control Antibody, catalog number: 401404, Biolegend). Immunoreactivity of indicated antibodies was determined by incubation with DAB substrate (MACH 2 Detection Kit, Biocare Medical) and counterstained with hematoxylin.

### *In Vitro* Expansion of γδ T Cells

BLT huMice were sacrificed at 4-6 weeks post HIV/mock infection, and fully developed lymphoid tissues were collected, and single cells were isolated following mechanical dissociation. Homogenized spleen and thymus tissues were passed through a 100um filter to obtain single-cell suspensions. Red blood cells were lysed and removed from spleen samples using ACK Lysing Buffer (ThermoFisher) as described by the manufacturer. Cells isolated from the BLT huMice splenocytes were cocultured with allogeneic monocytes (4:1 ratio) from HIV-seronegative human blood bank donors in the presence of nitrogen-containing bisphosphonate zoledronate (ZOL, 5uM) (Zoledronic Acid, Selleckchem, S1314) and recombinant human (rh)IL-2 (Proleukin, 100 IU/mL; Prometheus Laboratories) for ten days as previously described (16). rhIL-2 (100 IU/ml) was subsequently added every three days. The ten-day-cultured γδ T cells were characterized by flow cytometry analysis.

### HIV Infection of BLT HuMice

X4-tropic HIV lab strain NL4-3 (17, 18) was generated by transfection of 293T cells (ATCC; ATCC CRL-3216) with a plasmid containing a full-length HIV genome and collecting the HIV containing culture supernatant. The viral titer was determined by HIV-1 p24 AlphaLISA Assay (PerkinElmer, cat. No. AL291F) as described in the manufacturer’s protocol (19). Supernatant from uninfected 293T cells was used as a mock control. BLT huMice were anesthetized at 20-22 weeks post-transplantation and inoculated with mock control supernatant or HIV-1 (~1 × 10^5^ infectious units) by i.v. Injection *via* retroorbital delivery.

### HIV-1 Genomic RNA Detection

Total RNA was purified from plasma using RNA-Bee (AMSBIO). The RNA was then reverse-transcribed using TaqMan Reverse Transcription Reagents (Invitrogen) and quantitatively detected by real-time PCR using the TaqMan Universal PCR Master Mix (Invitrogen) with primers (forward primer, 5′ - CCCATGTTTTCAGCATTATCAGAA - 3′, and reverse primer, 5′ - CCACTGTGTTTAGCATGGTGTTTAA - 3′) and detection probe targeting HIV Gag gene (5′ - AGCCACCCCACAAGA - 3′) (20). The assay sensitivity/cutoff was ten copies/ml.

### Adoptive Transfer of T Cells to BLT HuMice

PBMC derived CD4^+^ T cells were isolated from HIV-positive individuals using EasySep Human CD4^+^ T Cell Isolation Kit and activated overnight with Human T-Activator CD3/CD28 Dynabeads (Life Technologies). The next day Dynabeads were separated from the CD4^+^ T cells by manual dissociation followed by magnet isolation. The activated CD4^+^ T cells were, washed, resuspended in PBS, and adoptively transferred into BLT huMice *via* intraperitoneal injection (5 million cells/100μl/mouse). PBMC from the allogenic HIV non-infected donor were cultured in the presence of ZOL and rhIL-2 for ten days to expand the Vδ2 cells. Activated and expanded Vδ2 cells were enriched using gamma delta T cells EasySep negative selection kit (Catalog-19255). This pure gamma delta T cells were adoptively transferred to BLT huMice *via* intraperitoneal injection (10 million/100μl/mouse) at the same time point when CD4^+^ T cells were injected. The BLT huMice were divided into two treatment cohorts; one that received only activated CD4^+^ T cells from HIV-infected donor, and the other that received the activated HIV-infected CD4^+^ T cells as well as *in vitro* expanded allogenic Vδ2 cells.

### Statistics

Differences between HIV-infected/uninfected humans and BLT huMice were compared using the two-tailed unpaired Student t-test. Differences among the human or BLT huMice groups were compared using the two-tailed paired students t-test. The normality of the samples was tested using the Shapiro-Wilk normality test. Statistical analyses were performed using the Prism8 (GraphPad Software), and p values <0.05 were considered statistically significant. The sample numbers and statistical analyses used are specified in each figure legend.

### Use of Human Fetal Tissue and Biological Agents

We described the approval of the use of human fetal tissue and biological agents in the previous study (21). Briefly, human fetal liver and thymus (gestational age of 18–20 weeks) were obtained from medically, or elective indicated termination of pregnancy through Magee-Women’s Hospital of UPMC *via* the University of Pittsburgh, Health Sciences Tissue Bank, or Advance Bioscience Resources Inc. Written, informed consent of the maternal donors was obtained in all cases, under IRB of the University of Pittsburgh guidelines and federal/state regulations. See details in the “Human Ethical Approval and Informed Consent” section.

### Approval for Using Animals and Biological Agents for *In Vivo* Experiments

The use of biological agents (e.g., HIV), recombinant DNA, and transgenic animals was reviewed and approved by the Institutional Biosafety Committee (IBC) at the University of Pittsburgh. All animal studies were approved by the IACUC at the University of Pittsburgh and were conducted following the NIH guidelines for housing and care of laboratory animals as well as the ARRIVE guidelines 2.0 for reporting of *in vivo* experiments involving animal research (22).

### Human Ethical Approval and Informed Consent

The study was performed following the guidelines of “Ethical Principles for Medical Research Involving Human Subjects” provided by the World Medical Association Declaration of Helsinki (1964) and its subsequent amendments (23). Written informed consents were obtained from the human study participants from the Pittsburgh Men’s Study, Multicenter AIDS Cohort Study (PMS-MACS) and the maternal donors of fetal tissues used in the study following the University of Pittsburgh IRB guidelines as well as federal/state regulations. The ethical use of human fetal organs/cells to perform the studies was reviewed before study initiation by the University of Pittsburgh IRB, which determined that the submitted study does not constitute human subject research as defined under federal regulations [45 CFR 46.102 (d or f) and 21 CFR 56.102(c), (e), and (l)]. The ethical use of human hematopoietic stem cells was reviewed and approved by the University of Pittsburgh Human Stem Cell Research Oversight (hSCRO) committee.

## Results

### Reconstitution of Human γδ and αβ T Cells in BLT HuMice

**Figure 1:**
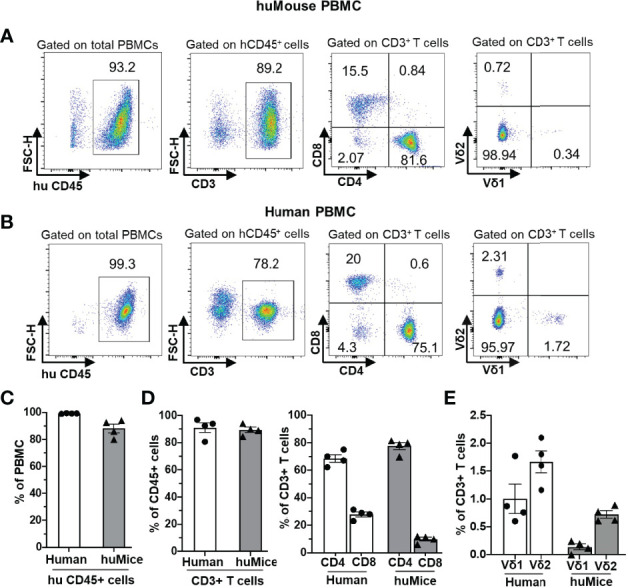
Human αβ and γδ T cell development in the peripheral blood of BLT huMice. **(A, B)** Representative flow cytometry analysis of human immune cell (hCD45^+^) reconstitution along with lymphocytes subsets, including αβ T cells (CD3^+^), (CD4^+^), (CD8^+^), and γδ T cells (Vδ1 and Vδ2 T cell subsets) in PBMC of BLT huMice **(A)** at ten weeks after transplantation and uninfected human **(B)**. **(C–E)** Quantification of human CD45^+^ lymphocytes **(C)**, human αβ **(D)**, and γδ **(E)** T cell subsets in PBMCs of BLT huMice and healthy humans (n = 4 biological replicates each).

**Figure 2:**
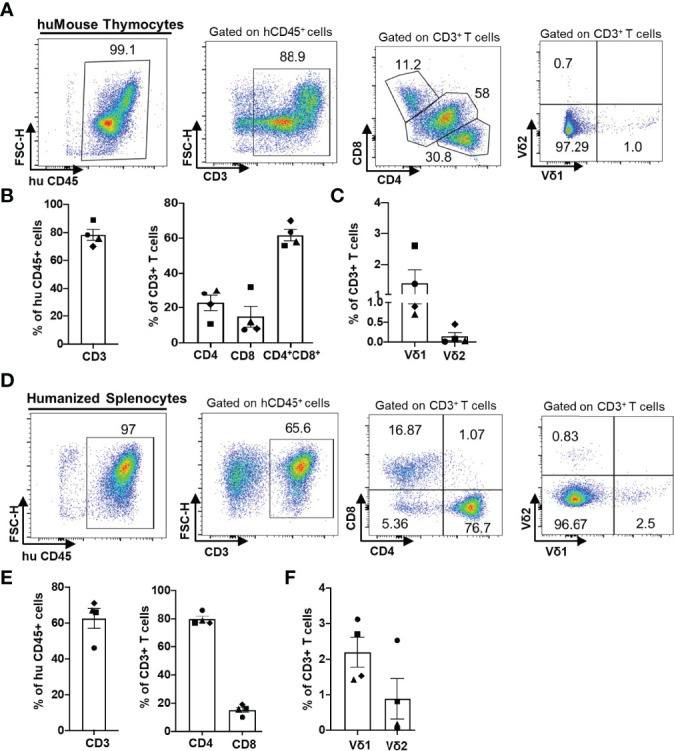
Human αβ and γδ T cell development in lymphoid tissues of a BLT huMouse model. **(A, D)** Representative flow cytometry analysis of human immune cell (hCD45^+^) reconstitution along with lymphocytes subsets including αβ T cells (CD3^+^), (CD4^+^), (CD8^+^) and γδ T cells (Vδ1 and Vδ2 T cell subsets) in lymphoid tissue [thymus **(A)** and murine spleen **(D)**] of BLT huMice **(A)** at 22 weeks post-transplantation. Quantification of human αβ and γδ T cells in the engrafted human thymus **(B, C)** and murine spleen tissue **(E, F)** of BLT huMice at 22 weeks post-transplantation (n = 4 biological replicates).

We first examined the reconstitution of human αβ and γδ T cells in huMice using multicolor flow cytometry (**Figure 1**). Importantly, when denoting γδ T cells in our study, we are referring only to the Vδ1 and Vδ2 T cell subtypes, which together account for ~98% of the total γδ T cell population in human blood (24, 25). We validated the flow cytometry assay for detecting human γδ T cells by demonstrating the presence of γδ+ cells in the CD3+ population and the absence of γδ+ cells in the CD3- population of human CD45+ cells from human peripheral blood (**Supplementary Figures S1A–D**). The gating scheme is shown for a representative sample of PBMC derived from a BLT huMouse (**Figure 1A**). We compared these results to PBMC samples from HIV seronegative humans, with data from a representative donor is shown in **Figure 1B**. We observed a high level of reconstitution of human CD45^+^ cells (~90%) in the peripheral blood of BLT huMice (**Figure 1C**). Approximately 90% of these human CD45^+^ cells were CD3^+^ T cells, of which, on average, were comprised of 80% CD4^+^ T cells and 15% CD8^+^ T cells (**Figure 1D**). This CD4/CD8 ratio was slightly higher than what is typically seen in humans, as shown with the four donors we tested that displayed a mean of 70% CD4^+^ T cells and 30% CD8^+^ T cells (**Figure 1D**). We also analyzed the γδ T cell subsets present in the peripheral blood of BLT huMice and determined a mean of 0.3% and 0.7% of total CD3^+^ T cells being comprised of Vδ1 T cells and Vδ2 T cells respectively (**Figure 1E**). The relative frequencies of these two subsets are comparable to, albeit lower than, the γδ lymphocyte populations found in the peripheral blood of healthy humans represented in our analysis showing 1% and 1.6% of total CD3^+^ T cells being Vδ1 and Vδ2 T cells, respectively (**Figure 1E**). To our knowledge, this is the first report to describe the reconstitution of human γδ T cells in BLT huMice. We also examined human immune cell populations reconstituted in the engrafted human thymus and murine spleen of each BLT huMouse (**Figure 2**). The gating scheme is shown for a representative sample of immune cells isolated from the human thymus (**Figure 2A**) and murine spleen (**Figure 2D**). Of the human CD3^+^ T cells isolated from the thymic tissue, an average of 22% were CD4^+^ T cells, 16% were CD8^+^ T cells, and 60% had an immature T cell phenotype being positive for both CD4 and CD8 (CD4^+^/CD8^+^, double-positive) (**Figure 2B**). In the murine splenic tissue, on average, the total T cell population comprised 80% CD4^+^ T cells and 16% CD8^+^ T cells (**Figure 2E**). Human γδ T cell subsets (Vδ1 and Vδ2) were also detected in these lymphoid tissues. From the human thymus, an average of 1.5% of the T cells had a Vδ1 cell phenotype, and 0.2% were Vδ2 T cells (**Figure 2C**). We observed a slightly higher prevalence of γδ T cell subsets isolated from murine spleen tissue, with a mean of 2.2% and 0.9% of the total T cell fraction consisting of Vδ1 T cells and Vδ2 T cells respectively (**Figure 2F**). We observed that Vδ2 T cells were predominantly present in the peripheral blood of BLT huMice (**Figure 1E**), while Vδ1 T cells were present primarily in the lymphoid tissues of BLT huMice (**Figures 2C, F****)**. The murine spleen of the BLT huMouse (hereafter referred to as the “humanized spleen”) had an approximate 2-fold higher reconstitution of γδ T cells than what was found in the thymus. This overall distribution of γδ T cell subsets (Vδ1 and Vδ2) in BLT huMice is comparable to those in human peripheral blood and tissue (26, 27). Lastly, we validated the flow cytometry-based detection of human γδ T cells in the lymphoid tissues in the BLT huMouse model *via in-situ* detection using immunohistochemistry (**Supplementary Figure S2**). In summary, these findings demonstrated that BLT huMice sustains physiologically relevant proportions of human αβ and γδ T cells in the periphery, engrafted human thymus, and (humanized) murine spleen.

### HIV Infection Alters γδ T Cell Populations in BLT HuMice and Humans

**Figure 3:**
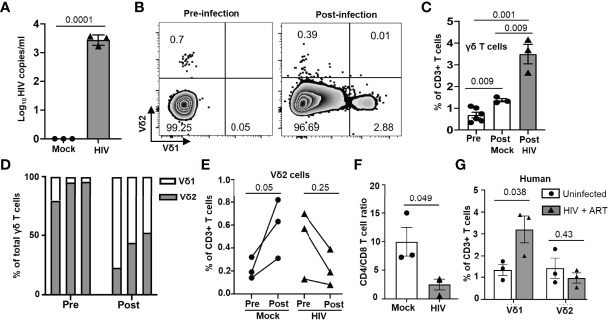
Peripheral blood γδ T cell number is altered in HIV-infected BLT huMice and humans. **(A)** HIV-1 replication (HIV RNA genome copies per ml) in the blood following HIV_NL4-3_ inoculation at 1X 10^5^ IU per mouse measured by qPCR (n = 3 biological replicates per group). **(B)** Representative flow plot showing the change in frequency of peripheral blood γδ T cell subsets before and after HIV infection. **(C)** Frequency of total γδ T cells before and after HIV infection in mock and HIV-infected BLT huMice analyzed by flow cytometry (n = 3 biological replicates per group). **(D)** Graphical representation of the change in frequencies of Vδ1 and Vδ2 cells within γδ population before and two weeks after infection. **(E)** Quantitation of changes in Vδ2 T cell frequency before and two weeks after infection in peripheral blood of HIV-infected and non-infected BLT huMice. **(F)** Comparison of changes in CD4^+^/CD8^+^ T cell ratio in peripheral blood of HIV-infected and non-infected BLT huMice analyzed by flow cytometry. **(G)** The frequency of Vδ1 and Vδ2 T cell subsets in the peripheral blood of ART-treated HIV-positive and HIV-negative individuals were analyzed by flow cytometry. Data are presented as a mean value ± SEM. P values <0.05 were considered statistically significant. P values were determined using paired 2-tailed Student’s t-test for comparing changes in γδ T cells population within the same cohort at two different time points, whereas an unpaired, 2-tailed Student’s t-test was used to compare differences between 2 groups.

To investigate the impact of HIV infection on γδ (referring to Vδ1 and Vδ2 T cell subsets) and αβ T cell populations, we infected BLT huMice with a laboratory strain of HIV-1_NL4-3_. Consistent with the previous studies, HIV RNA copies were detected in the peripheral blood of the HIV-infected BLT huMice as early as two weeks post-infection (**Figure 3A**) (12, 21, 28). PBMC from mock-inoculated and HIV-infected BLT huMice were collected before and after HIV infection for further viral load analysis, and these mice were sacrificed for tissue collection four weeks after infection. We first determined the proportion of γδ T cells present in PBMC of HIV-infected and mock-infected BLT huMice before and after HIV infection. Representative flow cytometry analysis plots displaying the percentage of γδ T cells present at pre-and post-infection time points are shown in **Figure 3B**. The total proportion of γδ T cells increases in both mock-infected [p=0.009] and HIV-infected BLT huMice (p=0.001) compared to pre-infection levels, but HIV-infected BLT huMice exhibited 2.3-fold higher levels of total γδ T cells when compared to mock-infected BLT huMice (p=0.009) **(** **Figure 3C****)**. We further examined γδ T cell subsets and found that Vδ2 T cell proportions were higher before infection and lowered following infection in BLT huMice **(** **Figure 3D****)**. The altered proportion of γδ T cell subsets may be at least partially explained by the depletion of Vδ2 T cells in HIV-infected BLT huMice, though our values did not reach statistical significance (p=0.25) **(** **Figure 3E****)**. Contrary to HIV-infected BLT huMice, mock-inoculated BLT huMice exhibited an increase in Vδ2 T cell levels in the blood **(** **Figure 3E****)**, which suggests that Vδ2 cells are depleted in HIV infection. Furthermore, depletion of peripheral blood CD4^+^ T cells in HIV-infected BLT huMice significantly decreases the CD4^+^/CD8^+^ T cell ratio (p =0.049) (**Figure 3F**). These results are consistent with what has been previously reported in human γδ T cell studies (29, 30). We observed similar γδ T cell trends in PBMCs isolated from healthy and ART treated HIV-positive individuals; wherein HIV-positive donors with ART had higher Vδ1 T cell levels and slightly lower Vδ2 T cell levels than healthy donors **(** **Figure 3G****)**.

**Figure 4:**
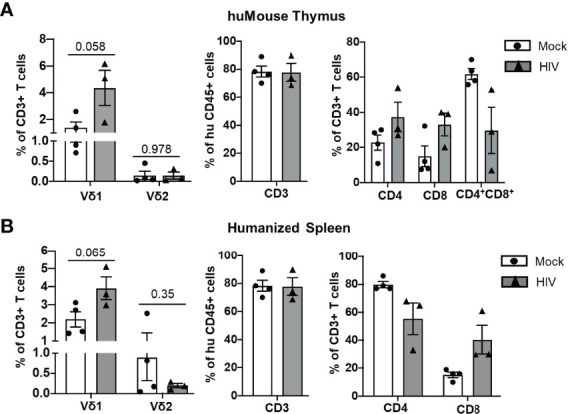
T cell number is altered in lymphoid tissue of HIV-infected BLT huMice. **(A, B)** Quantification of human T cell subsets, γδ T cells and αβ T cells in human thymus and humanized spleen tissue of HIV-infected (n = 3 biological replicates) and non-infected (n = 4 biological replicates) BLT huMice at 4-6 weeks post-infection. Data are presented as mean values ± SEM. P values <0.05 were considered statistically significant as determined using an unpaired, 2-tailed Student’s t-test.

In HIV-positive humans, lymphoid tissues are known to be sanctuaries for the latent HIV reservoir during ART (31). Therefore, we assessed the impact of HIV infection on the lymphocytes derived from lymphoid tissues of BLT huMice by flow cytometry analysis. A representative gating strategy used for this analysis is shown in **Figure 2**. Although not statistically significant, we observed an approximately 3-fold increase in the frequency of Vδ1 T cells in the human thymus (p = 0.058), and an approximately 2-fold increase in the humanized spleen of HIV-infected BLT huMice (p = 0.065) when compared to respective tissues from mock-infected BLT huMice (**Figures 4A, B**, **Supplementary Figures S3A, B**). This suggests that the frequency of Vδ1 T cells is increased in the lymphoid tissue of BLT huMice during HIV infection. We did not find a significant difference between the Vδ2 T cell population frequencies derived from the lymphoid tissues of HIV-infected or mock-infected BLT huMice. Besides γδ T cells, we found approximately a 2-fold increase in the proportion of CD8+ T cells derived from thymus and humanized spleen tissue of HIV-infected BLT huMice as compared to the mock-inoculated mice, suggesting a rapid proliferation of cytotoxic T cells in response to HIV infection (**Figures 4A, B**, **Supplementary Figures S3C, D**).

### HIV Infection in BLT HuMice and Humans Impairs Vδ2 T Cells Responsiveness to Stimuli

**Figure 5:**
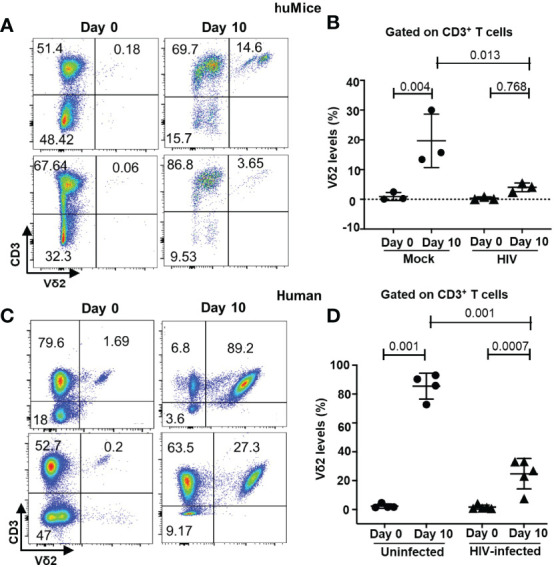
HIV infection impairs the *ex vivo* expansion of Vδ2 T cells. **(A, B)** BLT huMice were sacrificed at 4-6 weeks post-HIV/mock infection, and splenocytes isolated from the humanized spleen of BLT huMice were cultured in the presence of zoledronate IL-2, and uninfected allogenic monocytes (n = three mice per group). **(C, D)** Flow plots represent *in vitro* expansion of Vδ2 cells from HIV-infected and non-infected individuals in the presence of zoledronate and IL-2. Expansion of Vδ2 cell frequency was significantly higher in HIV-negative donors (n = 4) compared to HIV-positive donors (n = 5 biological replicates). Data are presented as mean values ± SEM. P values <0.05 were considered statistically significant as determined using a 2-way ANOVA test.

To demonstrate the *ex vivo* responsiveness of Vδ2 T cells to activation factors and their potential for therapeutic evaluation, we cultured leukocytes derived from splenocytes of BLT huMice (n = 6), peripheral blood of ART-suppressed HIV-positive (*n* = 5), and age-matched HIV-negative individuals (*n* = 4) and stimulated them with the combination of ZOL and recombinant human Interleukin-2 (rhIL-2). The basal percentage of Vδ2 cells within the CD3^+^ population of lymphocytes was analyzed by flow cytometry, which revealed a range of inter-individual differences among HIV-negative donors (1.2% - 2.2%), ART-suppressed HIV-positive individuals (0.5% - 1.2%), and BLT huMice (0.2% - 1%). Initially, when we cultured Vδ2 T cells from the peripheral blood or the lymphoid tissues of BLT huMice in the presence of ZOL and rhIL-2, we observed modest expansion of Vδ2 T cells, but it was not optimal. Next, we supplemented the cultures with allogenic monocytes from healthy individuals and obtained higher expansion of Vδ2 T cells. Our results show that Vδ2 T cell expansion from splenocytes of mock-inoculated BLT huMice after ten days was approximately 4-fold higher than HIV-infected BLT huMice (p=0.013) (**Figures 5A, B****)**. Similarly, we expanded Vδ2 T cells from HIV-positive and HIV-negative individuals and found that Vδ2 T cell expansion was approximately 3-fold higher in HIV-negative individuals than HIV-positive individuals (p=0.001) (**Figures 5C, D****)**. These results suggest that HIV infection not only reduces the frequency of Vδ2 T cells *in vivo* but it also adversely impacts the ability of these cells to expand in response to stimuli.

### The Phenotype of Ex-Vivo Expanded Vδ2 T Cells

**Figure 6:**
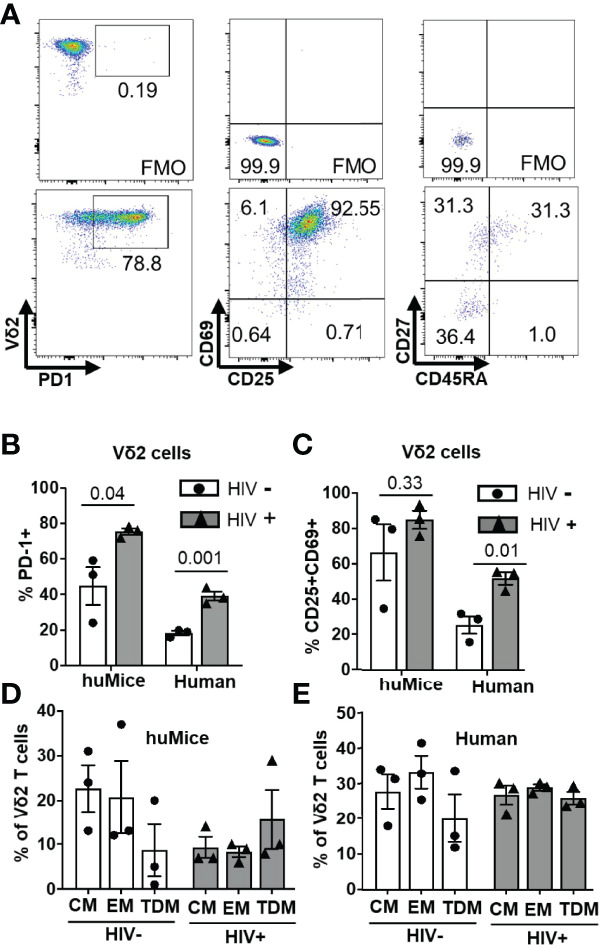
Phenotypic characterization of cultured Vδ2 cells. The phenotype of Vδ2 cells from 6 BLT huMice and 6 HIV-positive/negative individuals after the expansion was analyzed by flow cytometry. **(A)** Representative flow cytometry analysis of expanded Vδ2 cells from splenocytes of humanized mice expressing activation, inhibitory, and differentiation markers. **(B)** Expression of the checkpoint inhibitory marker PD-1 on Vδ2 cells expanded from HIV-infected and non-infected BLT huMice and humans. **(C)** Dual expression of activation markers CD69 and CD25 on Vδ2 cells expanded from HIV-infected and non-infected BLT huMice and humans. **(D)** Percentage of Vδ2 cells defined as central memory (CM) (CD45RA^–^CD27^+^), terminally differentiated (TDM) (CD45RA^+^ CD27^–^), and effector memory (EM) (CD45RA^–^CD27^–^) derived from HIV-infected and non-infected BLT huMice. **(E)** Percentage of Vδ2 cells derived from HIV-positive and HIV-negative individuals defined as having EM, CM, TDM phenotypes. Data are presented as mean values ± SEM. P values were determined using two-tailed unpaired t-tests between the two groups.

The phenotype of expanded Vδ2 cells after ten days of exposure to ZOL and rhIL-2 was analyzed in a subgroup of HIV-positive/HIV-negative individuals and HIV-infected/uninfected BLT huMice by measuring the expression of markers of activation and differentiation by flow cytometry (**Figure 6A**). Surface expression of the inhibitory receptor PD-1 was observed in a mean of 78% and 45% on the cultured Vδ2 cells derived from HIV-infected and uninfected BLT huMice, respectively (p=0.04) (**Figure 6B**). Similarly, the mean percentage of Vδ2 cells expressing PD-1 from HIV-positive and HIV-negative human donors was respectively 40% and 20% (p=0.001) (**Figure 6B**). The activation markers CD69 and CD25 were co-expressed on a mean of 80% and 65% of the Vδ2 cells cultured from HIV-infected and uninfected BLT huMice, respectively. Similarly, CD69 and CD25 co-expression was observed in a mean of 50% and 25% of the Vδ2 T cells from HIV-positive and HIV-negative human donors, respectively (**Figure 6C**). Together, these findings suggest that the expression of activation markers on Vδ2 cells expanded *in vitro* are slightly higher in those derived from HIV-positive humans and BLT huMice than from their HIV-negative counterparts. We also evaluated the differentiation status of the cultured Vδ2 cells based on memory cell phenotypes defined as follows: (CM) central memory (CD45RA^–^CD27^+^), (TDM) terminally differentiated (CD45RA^+^CD27^–^) and (EM) effector memory (CD45RA^–^CD27^–^). Although not statistically significant, we noted an increase in the TDM phenotype and a decrease in the CM and EM phenotypes in the *in vitro* expanded Vδ2 T cells derived from HIV-infected BLT huMice compared to the Vδ2 cells cultured from uninfected BLT huMice (**Figure 6D**). However, in humans, we found an approximately equal distribution (20-30%) of EM, CM, TDM phenotypes between HIV-positive and HIV-negative individuals (**Figure 6E**).

### Adoptive Transfer of Vδ2 T Cells Did Not Control Cell-Associated HIV Transmission and Replication in BLT HuMice

Many *in vitro* studies have demonstrated a protective role of γδ T cells against HIV infection (8, 9, 32). Therefore, we tested the impact of adoptively transferred allogeneic Vδ2 cells in an *in vivo* model of cell-associated HIV transmission and replication using BLT huMice, which is physiologically relevant to HIV transmission in humans. As discussed above, *in vitro* expansion of Vδ2 T cells from HIV-infected individuals was not optimal. We overcame this limitation in our adoptive transfer experiment by utilizing Vδ2 T cells expanded from allogeneic non-infected individuals. A similar strategy was previously demonstrated to be safe and effective in humans (33). Moreover, it is therapeutically relevant because Vδ2 T cells lack functional MHC restriction and pose a minimal risk for developing graft-versus-host complications (34). However, they may serve as targets for an allogeneic response by the engrafted immune cells, albeit graft versus host disease (i.e., alopecia) was not observed during the short duration of this experiment.

**Figure 7:**
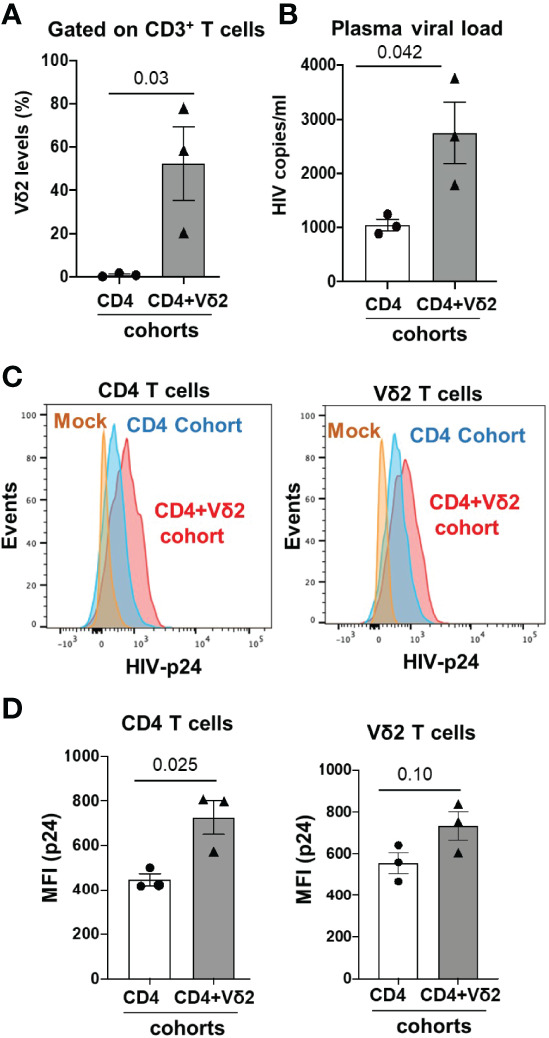
Adoptive transfer of Vδ2 T cells increases HIV replication in cell-associated HIV transmission in BLT huMice. **(A)** Vδ2 cell number significantly increased 2-weeks post-adoptive transfer in peripheral blood of BLT huMice (n = 3 biological replicates per group); analyzed by flow cytometry. **(B)** HIV viral load increased significantly in plasma of Vδ2+CD4-engrafted BLT huMice compared to CD4-engrafted BLT huMice; measured *via* qPCR at two weeks post-adoptive transfer (n = 3 biological replicates per group). **(C)** Representative flow cytometry histogram plots of peripheral blood total CD4^+^ T cells and Vδ2 cells expressing HIV p24 respectively. **(D)** HIV p24 is slightly higher in peripheral blood total CD4^+^ T cells and Vδ2 T cells of BLT huMice that received CD4+Vδ2 treatment compared to the BLT huMice that received only CD4^+^ T cells treatment respectively (n = 3 biological replicates per group). Data are presented as mean values ± SEM. P values were determined using a two-tailed paired t-test within the treatment groups.

BLT huMice were grouped into two different cohorts: one cohort received only activated CD4^+^ T cells from an HIV-infected human donor (CD4-only cohort) to mimic cell-associated HIV transmission (13, 14), while the other cohort received simultaneous injections of activated CD4^+^ T cells from an HIV-infected human donor and cultured activated allogenic Vδ2 cells from an uninfected human donor (CD4+Vδ2 cohort). Before the adoptive transfer of CD4 and Vδ2 T cells, we assessed the human immune cell reconstitution in all the BLT huMice, and we observed approximately similar levels of huCD45^+^ cells and huCD4^+^ T cells in all the BLT huMice **(** **Supplementary Figure S4****)**. Reconstitution of human Vδ2 and CD4^+^ T cells in the peripheral blood of BLT huMice was examined *via* flow cytometry two weeks after the adoptive transfer procedure. We found that a mean of 50% of all CD3^+^ T cells was Vδ2 T cells in the peripheral blood of CD4+Vδ2 cohort, whereas less than 1% of all CD3^+^ T cells were Vδ2 T cells in CD4-only cohort (p=0.03) (**Figure 7A**), which indicated successful engraftment of human Vδ2 T cells in the BLT huMice. Next, we confirmed HIV replication in the plasma of BLT huMice by qPCR two weeks after adoptive transfer. Surprisingly, we observed a viral load in the CD4+Vδ2 cohort was approximately 2-fold higher than the CD4-only cohort (**Figure 7B**) (p=0.042). Hypothesizing that this increase in viral load could be due to HIV-infection of the adoptively transferred Vδ2 T cells, we decided to analyze the CD4^+^ T cells and Vδ2 T cell subsets in the peripheral blood of both cohorts at two weeks post-adoptive transfer. Representative flow cytometric plots of HIV p24 levels in total CD4^+^ T cells and Vδ2 T cells from both cohorts are shown in **Figure 7C**. We observed a slightly higher presence of HIV p24 in total CD4^+^ T cells (p=0.025) and Vδ2 T cells (p=0.10) in the CD4+Vδ2 cohort of BLT huMice compared to the reference CD4-only cohort (**Figure 7D**). Therefore, the adoptive transfer of Vδ2 T cells appears to exacerbate HIV replication in BLT huMice (**Supplementary Figure S5**). Moreover, *in vitro* co-culture of HIV-infected CD4^+^ T cells with Vδ2 T cells also suggests that in the presence of Vδ2 T cells, HIV infection increased, and they failed to limit the viral replication (**Supplementary Figure S5**). Additionally, we analyzed the blood and lymphoid tissue associated viral load at four weeks post-adoptive transfer and found no significant difference in the viral levels, which suggests that viral replication plateaued at this time point in BLT huMice of both the cohorts **(** **Supplementary Figure S6****).**

**Figure 8:**
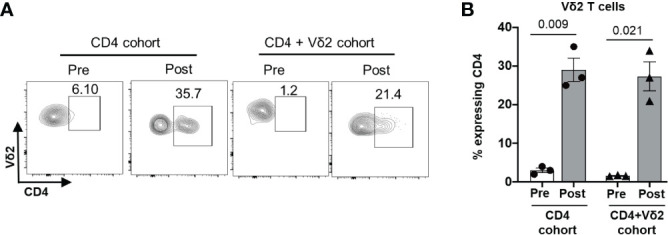
Induction of CD4 expression on Vδ2 T cells *in vivo* during HIV infection. CD4^+^ T cells from an HIV-positive individuals were administered to BLT huMice with or without co-transfer of *in vitro* activated Vδ2 T cells. **(A)** Representative flow cytometry analysis of CD4 expression on Vδ2 T cells before and after cell transplant. **(B)** CD4 expression on human Vδ2 T cells from the BLT huMice was measured by flow cytometry analysis pre-and post- (2 weeks) cell transplant. Data are presented as mean values ± SEM. P values were determined using a two-tailed paired t-test within the treatment groups.

Despite the low or lack of CD4 receptor expression on Vδ2 T cells, our *in vivo* data suggest that these cells can be targets of HIV infection. This is in accordance with a previous study from Sarabia et al., which reported that resting Vδ2 cells act as reservoirs for latent HIV infection (35). We posited that HIV infection could impact the phenotype of Vδ2 T cells to make them more susceptible to direct infection. Since Vδ2 T cells already express high levels of the CCR5 co-receptor, we examined whether the expression of the CD4 receptor on Vδ2 T cells was induced on this cell type during HIV infection. Before adoptive transfer, less than 5% of endogenous (**Figure 8A** and *in-vitro* cultured Vδ2 T cells **(** **Supplementary Figure S7****)** expressed the CD4 receptor, but at two weeks after adoptive transfer, we indeed detected a mean of 30% of Vδ2 T cells expressing the CD4 receptor in both the cohorts (**Figures 8A, B****)**. Contrary to the previous reports (9, 36) highlighting the protective function of Vδ2 T cells in controlling HIV infection *in vitro*, our result suggests that HIV infection can drive CD4 expression on Vδ2 T cells *in vivo*, priming them to become targets for HIV infection and contributors to viral dissemination.

## Discussion

γδ T cells are the first line of defense against many pathogens, but their frequency and functions are severely altered in the setting of many infectious diseases, including HIV (7). Despite long-term ART and viral control, γδ T cells do not reconstitute HIV-infected individuals to their levels set before infection (30). However, in HIV elite controllers, Vδ2 T cell numbers are maintained at normal levels throughout infection, implying that Vδ2 T cells play an essential role in HIV infection and control. Thus, a better understanding of Vδ2 T cells during HIV infection will be necessary to be effectively utilized or targeted for therapeutic benefit. While prior studies have demonstrated the protective effect of γδ T cells against HIV infection *in vitro* (8, 9, 32), there is a lack of information available and a gap in knowledge regarding their therapeutic potential *in vivo*.

In this study, we offer the first evidence that clinical trends of γδ T cell subpopulations (Vδ1 and Vδ2) before and after HIV infection can be modeled in BLT huMice. Immunodeficient NSG mice exhibited robust reconstitution of human immune cells, including γδ T cells, by 12 weeks post-engraftment of CD34^+^ human fetal liver cells and thymic tissues. Flow cytometric analysis of human T cell subsets revealed that CD4^+^, CD8^+^, Vδ1, and Vδ2 T cell levels in both the blood and lymphoid tissues of healthy BLT huMice were comparable to those seen in healthy humans. Furthermore, we observed high levels of viremia two weeks following HIV infection, an associated depletion of Vδ2 T cells, and an expansion of Vδ1 T cells in the peripheral blood of BLT huMice. These features have been previously reported in several clinical studies (37–39). Thus, BLT huMice may overcome some of the translational limitations in non-human primate SIV models, which include unremarkable changes in Vδ1/Vδ2 T cell ratios, otherwise common in HIV infection in humans. Our study demonstrating the *in vivo* reconstitution of Vδ2 T cells in the BLT huMouse model also provides a proof-of-concept and basis for the design of future *in vivo* studies that further evaluate the role of human γδ T cells in the setting of HIV infection as well as other chronic diseases such as cancer.

Current HIV cure strategies utilize the effector functions of conventional CD8+ cytotoxic T cell lymphocytes (CTL) to kill the HIV-infected cellular reservoir following the induction of latency reversal (40). Unfortunately, the need to specifically stimulate or target the activation of autologous HIV-antigen specific autologous CD8^+^ T cells *ex vivo* or *in vitro* on an individual MHC/peptide-specific level and the existence of HIV CTL escape variants within the latent reservoir has challenged the progress of this approach (41, 42). γδ T cells offer an attractive alternative to CTL as a potential therapeutic tool to mediate anti-HIV effector functions. Their lack of MHC restriction may provide added benefits by raising the threshold for HIV to achieve immune escape. Moreover, since they pose a reduced risk of inducing allogeneic graft rejection, they may be considered for application in allogeneic immunotherapy settings. A previous study has shown that γδ T cells mediate inhibition of HIV replication (2), but the natural scarcity of γδ T cells in tissues and circulation indicates that these cells would likely need to be expanded *ex-vivo* for them to have the intended therapeutic effect. Although there are numerous *in vitro* protocols for expanding γδ T cells from bulk PBMC, two major approaches can be considered for targeting γδ T cells for clinical translation. First, both Zoledronic Acid (ZOL) and rhIL-2 can be administered to directly increase the proliferation of endogenous Vδ2 T cells (43). The other approach would be ex-vivo activation and expansion of Vδ2 T cells for adoptive therapy. In the HIV setting, this approach is limited by the substantial loss of Vδ2 T cells during the early stages of the infection cycle, which fail to fully recover after ART initiation. An alternative would be to harvest Vδ2 T cells from healthy donors and expand them *in vitro* using ZOL and rhIL-2 for allogeneic delivery, as has been previously reported in human cancer clinical trials (33, 44) and non-human primate models (45). One of these cancer trials demonstrated that the adoptive transfer of haploidentical expanded Vδ2 T cells from relatives of cancer patients was safe and effective for achieving meaningful responses (33). We attempted to culture and expand Vδ2 T cells derived from PBMC and lymphoid tissue of BLT huMice using ZOL and rhIL-2. Unfortunately, while we could expand these BLT huMice derived cells *in vitro*, we could not collect and generate an adequate number to carry out *in vivo* studies using this method. However, when we supplemented the cultures with allogeneic monocytes from healthy individuals to enhance ZOL-induced phosphoantigen presentation, we achieved a 20-fold increase in Vδ2 T cell expansion. Importantly, this was the first reported evidence that Vδ2 T cells derived from the splenocytes of BLT huMice can indeed be expanded *in vitro*.

Our pilot study examined the therapeutic potential of adoptively transferred Vδ2 T cells in HIV infection of BLT huMice during cell-associated HIV transmission using CD4+ T cells isolated from ART-treated HIV-positive individuals. Cell-associated HIV transmission is a widely reported means of HIV infection (13, 14). Furthermore, laboratory-derived molecular clones of HIV infection can exhibit different characteristics compared to naturally derived HIV strains (46). Although previous *in vitro* studies described the protective effect of γδ T cells against HIV infection (8, 9, 32), we did not see a therapeutic benefit, namely suppression of viremia, with the delivery of Vδ2 T cells in BLT huMice. Treatment with the activated Vδ2 cells resulted in higher viremia at two weeks post-infection as compared to the HIV-infected BLT huMice that were not co-engrafted with the Vδ2 T cells. Our findings were limited by the study’s sample size and low γδ T cell yield from the blood of BLT huMice, but future experiments will focus on the mechanisms of interactions between HIV and Vδ2 T cells. The ability to expand Vδ2 T cells from the murine spleen of BLT huMice provides an additional reservoir of cells for understanding HIV-associated activation or dysregulation of this cell type. Our adoptive transfer experimental data suggest that during the early stages of HIV infection, Vδ2 T cells can transiently upregulate the surface expression of CD4. This is in accordance with a previous study showing that the long-term culture of Vδ2 T cells in the presence of IL2 resulted in CD4 expression *in vitro* (35). Though we see a modest trend in our *in vitro* culture of Vδ2 T cells before the adoptive transfer, CD4 expression in Vδ2 T cells was unexpectedly more pronounced two weeks after transplantation into BLT huMice. While the mechanisms involved remain the subject of future studies, we speculate that this induction of CD4 expression on Vδ2 T cells may be a general inflammatory event in the host, triggered during some viral infections, a phenomenon which has also been noted to occur during COVID-19 infection (47). Moreover, expanded Vγ9Vδ2 T cells can produce pro-inflammatory cytokines that can potentially activate HIV replication. However, we cannot rule out the possibility that allogenic responses induced in the BLT huMice post-adoptive transfer may contribute to the increased HIV viremia associated with the delivery of Vδ2 T cells. Future methods may include introducing fluorescent tags to Vδ2 T cells and fetal HSCs before engraftment for tracking the proliferation, trafficking, or death of Vδ2 T cells during infection. Nevertheless, these findings raise more questions about the role of γδ T cells in the initial sequelae of HIV infection and their potential contribution to the HIV cellular reservoir, as has been previously reported (35).

To our knowledge, this is the first report demonstrating that functional human γδ T cells can be robustly reconstituted in a BLT huMice model. This small animal model provides a platform for future mechanistic studies to explore interactions between HIV and T cell subsets and, more broadly, for *in vivo* evaluation of γδ T cells and γδ T cell-based therapies in the setting of various human diseases.

## Data Availability Statement

The original contributions presented in the study are included in the article/**Supplementary Material**. Further inquiries can be directed to the corresponding authors.

## Ethics Statement

The animal study was reviewed and approved by Institutional Biosafety Committee (IBC) at the University of Pittsburgh.

## Author Contributions

SB, MB, and RM contributed to the experimental and study design. SB and YA performed the experiments. SB analyzed the data and prepared the manuscript. YA, RM, MB, and ML contributed to interpreting the results and critically edited the manuscript. All authors contributed to the article and approved the submitted version.

## Funding

This work was supported in part by NIH grant R01 AI152655-01A1.

## Conflict of Interest

The authors declare that the research was conducted in the absence of any commercial or financial relationships that could be construed as a potential conflict of interest.

## Publisher’s Note

All claims expressed in this article are solely those of the authors and do not necessarily represent those of their affiliated organizations, or those of the publisher, the editors and the reviewers. Any product that may be evaluated in this article, or claim that may be made by its manufacturer, is not guaranteed or endorsed by the publisher.
