# Supplementary material for: The BLT Humanized Mouse Model as a Tool for Studying Human Gamma Delta T Cell-HIV Interactions In Vivo
Source: Front Immunol. 2022 May 20;13:881607. doi: 10.3389/fimmu.2022.881607 (PMC9164110; doi:10.3389/fimmu.2022.881607)
Supplement: Supplementary file 1 [file DataSheet_1.pdf]

Supplementary figures

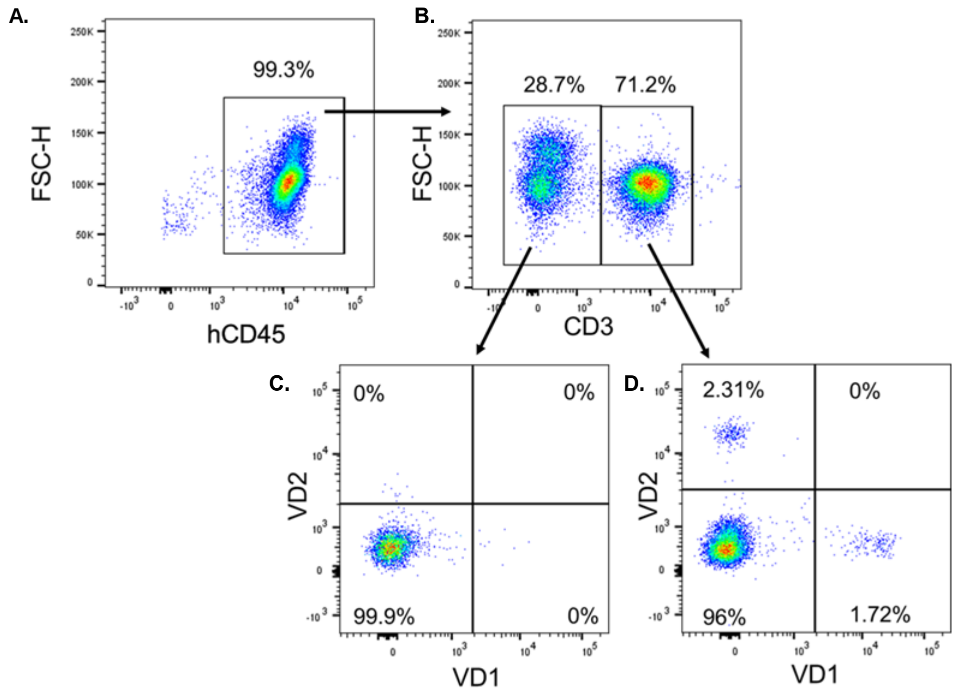

Supplementary Fig. S1. The specificity of human Vδ1 and Vδ2 T cells detection via flow cytometry.
Human CD45+ cells from human peripheral blood (A) were separated into CD3+ (T cells) and CD3- (non-T cells) cells (B) to determine the specificity of detecting human γδ T cell subsets (Vδ1 and Vδ2 T cells) via flow cytometry. We compared the expected absence of human Vδ1+ and Vδ2+ cells in human CD3- cells (non-T cells) (C) to the expected presence of human Vδ1+ and Vδ2+ cells in human CD3+ cells (T cells) (D).

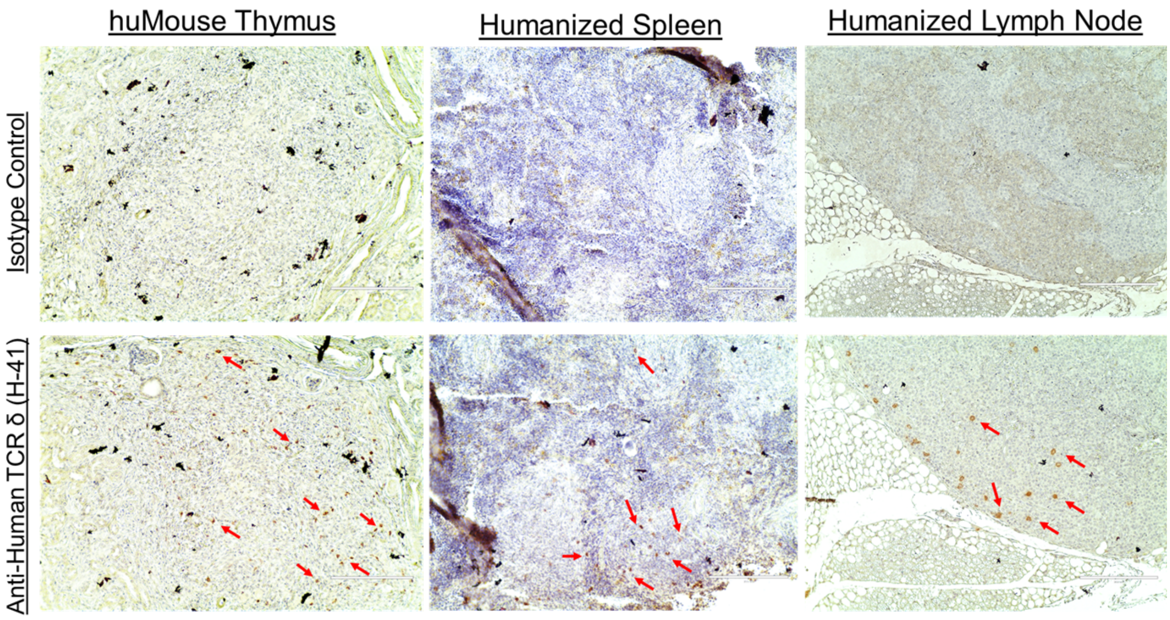

Supplementary Fig. S2. In-situ detection of human γδT cells in BLT huMice via immunohistochemistry.

Serial sections of thymus graft, humanized spleen and humanized lymph node in BLT huMice were stained via immunohistochemistry using anti-Human TCR δ (clone H-41) and isotope control antibodies. Images of the same region in the serial sections were taken for each tissue. Red arrows indicate the locations of Human TCR δ+ cells (dark brown stain; 3, 3'-diaminobenzidine (DAB) stain) in the sections.

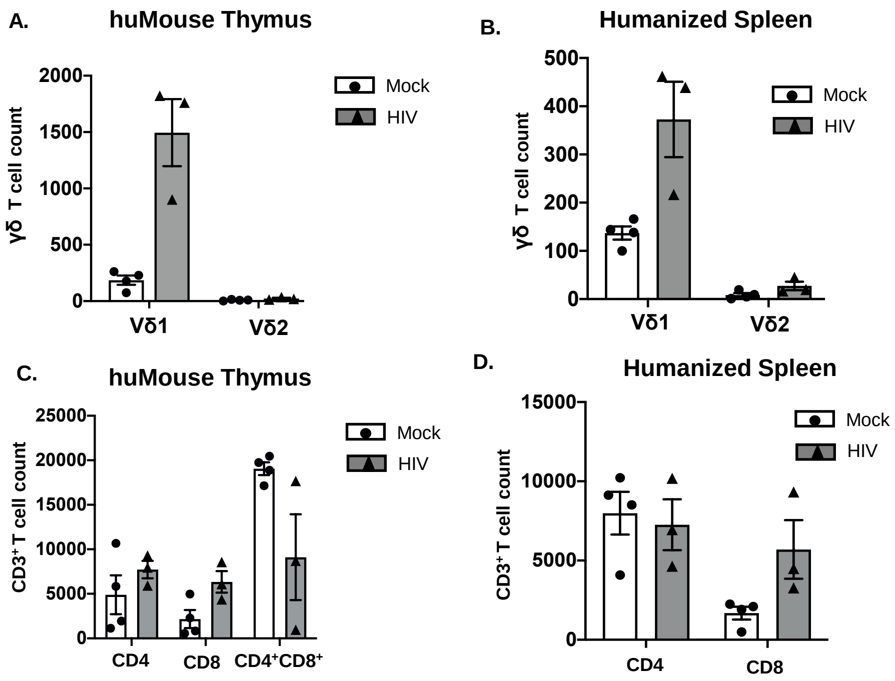

Supplementary Fig. S3. T cell number is altered in lymphoid tissue of HIV-infected BLT huMice.

(A-B) Quantification of human γδ T cell subsets (Vδ1 and Vδ2 T cells) in human thymus and humanized spleen tissue of HIV-infected (n=3) and non-infected (n = 4) BLT huMice at 4-6 weeks post-infection. (C-D) Quantification of human T cell subsets (CD4 and CD8 T cells) in human thymus and humanized spleen tissue of HIV-infected (n=3) and non-infected (n = 4) BLT huMice at 4-6 weeks post-infection

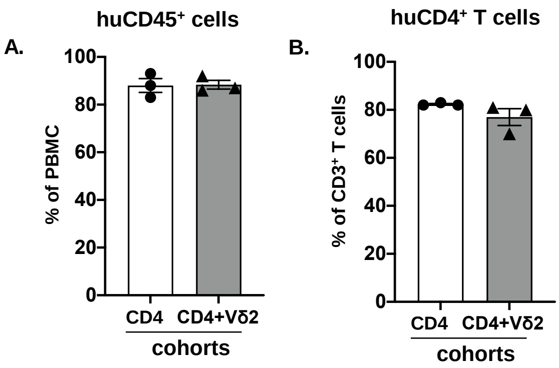

Supplementary Fig. S4. Reconstitution of human immune cells in treatment and experimental BLT huMice cohort is similar.

Quantification of human $CD45^+$ lymphocytes (A) and human $CD4^+$ T cells (B) in treatment and experimental BLT huMice cohort before adoptive transfer of human immune cells.

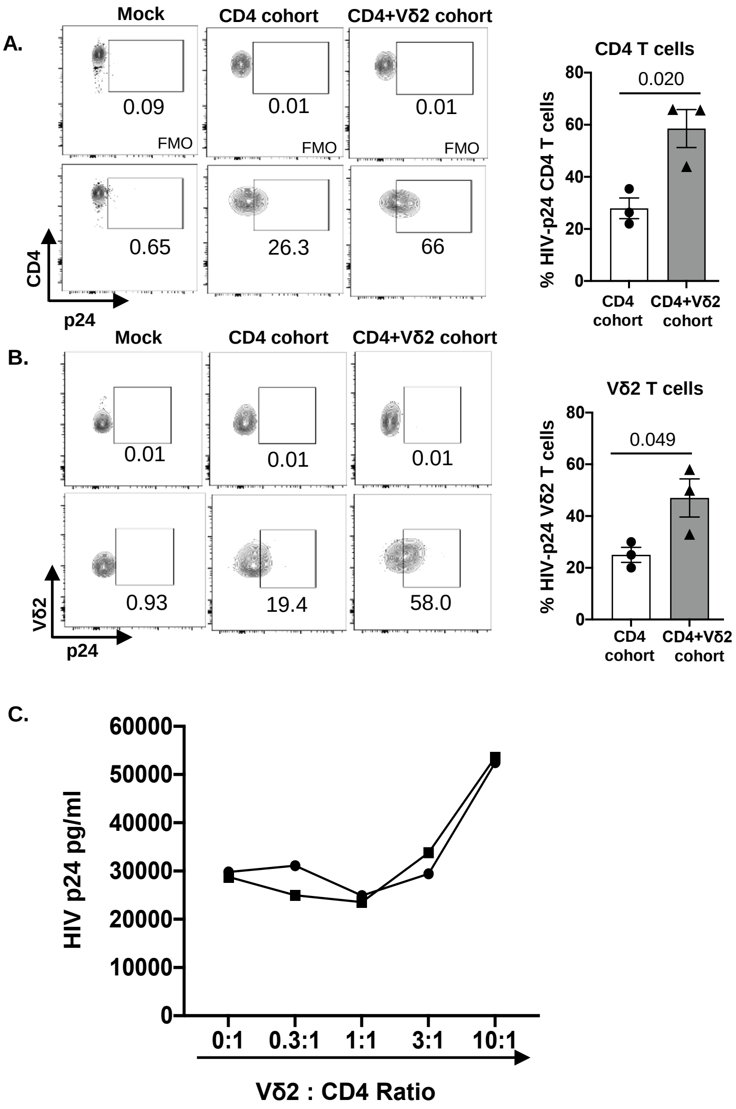

Supplementary Fig. S5. HIV infection is increased in the presence of Vδ2 T cells in BLT huMice and in vitro model.

(A) Representative flow cytometry plots of peripheral blood total CD4+ T cells that are expressing HIV p24 (n=3 per group). (B) Representative flow cytometry plots of peripheral blood total Vδ2 T cells that are expressing HIV p24 (n=3 per group). (C) $CD4^+$ T cells obtained from healthy individuals were infected with X4 tropic HIV NL4-3 and then co-cultured with zoledronate activated ex vivo expanded autologous Vδ2 T cells in various ratios for 7 days. Then supernatants were collected and analyzed for presence of HIV-p24 by ELISA (n=2).

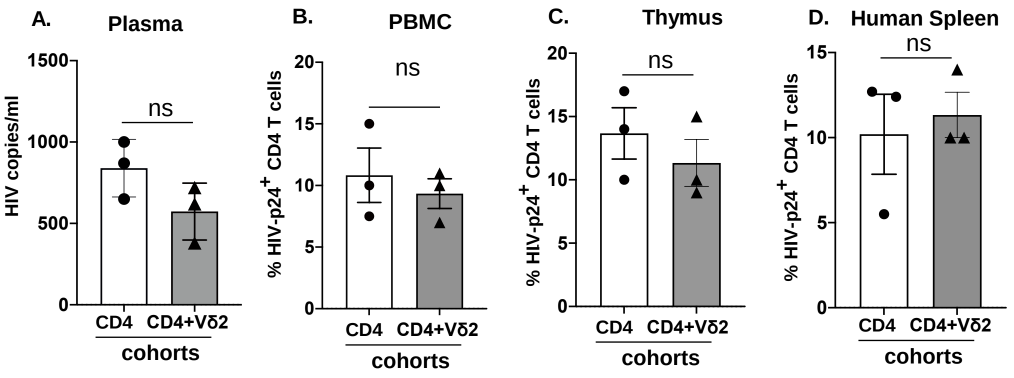

Supplementary Fig. S6. HIV viral load is similar in blood and lymphoid tissues of treatment experimental cohorts 4 weeks post cell transplant. Quantification of HIV viral load in plasma (A), $CD4^+$ T cells from PBMC (B), $CD4^+$ T cells from Thymus (C) and $CD4^+$ T cells from humanized spleen (D) of treatment (CD4+Vδ2 cohort) and experimental (CD4 cohort) BLT huMice cohorts (n= 3 per group) 4 weeks post cell transplant.

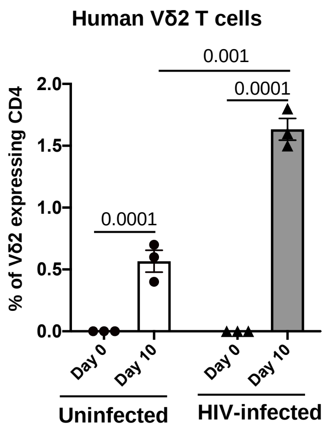

Supplementary Fig. S7. Induction of CD4 receptor on human Vδ2 T cells in an in vitro culture

PBMCs of HIV infected and uninfected individuals were cultured in the presence of zoledronate and IL-2 to induce *ex vivo* expansion of Vδ2 T cells. CD4 expression on Vδ2 cells was assessed at pre- and 10 days post-zoledronate and IL-2 treatment by flow cytometry (n=3 per group). Data are presented as mean values ± SEM. P values were determined using 2 tailed paired t-test within the treatment groups.
